# Data on the concentration of fractions and the total content of chemical elements in catenae within a small catchment area in the Trans Urals, Russia

**DOI:** 10.1016/j.dib.2019.104224

**Published:** 2019-07-05

**Authors:** Ivan Semenkov, Victoria Krupskaya, Galya Klink

**Affiliations:** a Lomonosov Moscow State University, Russia; b Institute of Geology of Ore Deposits, Petrography, Mineralogy and Geochemistry of the Russian Academy of Sciences, Russia; c Institute for Information Transmission Problems (Kharkevich Institute) of the Russian Academy of Sciences, Russia

**Keywords:** Agricultural soils, Mobile fractions, Rare earth elements, Trace elements, Potentially toxic elements, Mineralogy

## Abstract

Research on migration of chemical elements (ChEs) in soils is important for the understanding of geochemical processes in polluted and undisturbed landscapes. In this article, we report original data on Anthric Chernozems and Anthric Planosols within a small gully's catchment area in the Trans Urals (Russia). Mean total concentrations of 24 ChEs and content of mobile fractions (F1 – exchangeable, F2 – bound within organo-mineral complexes and F3 – bound with Fe and Mn hydroxides) of 61 ChEs including macro elements (Al, Ca, Fe, K, Mg, Mn, Na, P, Ti, S, Si), heavy metals (Ba, Co, Cr, Cu, Ni, Pb, Rb, Sr, Th, U, V, Zn), trace elements (Ag, As, B, Be, Bi, Br, Cd, Cs, Ge, Hf, Li, Mo, Nb, Pd, Sb, Sc, Se, Sn, Ta, Te, Tl, W, Zr) and rare earth elements (Ce, Er, Eu, Gd, La, Lu, Nd, Pr, Sm, Tb, Tm, Dy, Ho, Y, Yb) are determined from in a total of 60 samples from topsoil and subsoil of Anthric Chernozems and Anthric Planosols. The data obtained also include pH-value, total organic carbon content (TOC), seven particle-size classes (<2, 2–6.3, 6.3–20, 20–63, 63–200, 200–630 and 630–2000 μm), electrical conductivity and chemical composition (cations and anions) of water extracts as well as soil mineralogical composition.

**Table:** Specifications table

Subject area	*Environmental Chemistry, Earth Science*
More specific subject area	*Soil Science, Environmental Chemistry.*
Type of data	*Raw data, figures and tables*
How data was acquired	*Data were obtained by means of standard techniques*[1], [2], [3]*, an ‘Analizeter 22’ equipment (Germany), high performance liquid chromatography using a Styer chromatograph (Aquilon, Russia), an Axios X-Ray fluorescence spectrometer (made by PANalytical, Netherlands), an Elan-6100 ICP-MS System (Inductively Coupled Plasma Mass Spectrometer by PerkinElmer Inc., USA) and an Optima-4300 DV ICP-AES System (Inductively Coupled Plasma Atomic Emission Spectrometer by PerkinElmer Inc., USA), an ULTIMA-IV X-Ray diffractometer (made by Rigaku, Japan) with Cu radiation and a DTex/Ultra semiconductor detector.*
Data format	*Raw and analysed*
Experimental factors	*A total of 60 soil samples (300 – 500 g) were collected from the 11 pits (*Fig. 1, Fig. 2*) at the interfluve, slopes and bottom of a small gully on the Trans-Urals Elevation of the Ishim Plain (Western Siberia, Russia).*
Experimental features	*Samples for the particle-size distribution analysis were pre-treated with 4% Na*_*4*_*P*_*2*_*O*_*7*_*.* *Soil suspensions (*50 ml*) for mobile fraction extraction were prepared from soil subsamples (5–10 g) by incubation for 18 hours.*
Data source location	*The catchment area (480 000 m*^*2*^*) of a U-shaped gully on the Trans-Urals Elevation of the Ishim Plain (Russia) is occupied by Anthric Chernozems and Anthric Planosols. GPS coordinates of soil pits are:* *1. N 56°02′58,5″ E 63°35′00,4″* *2. N 56°02′56,8″ E 63°35′03,1″* *3. N 56°02′56,2″ E 63°35′04,8″* *4. N 56°02′55,7″ E 63°35′07,2″* *5. N 56°02′56,7″ E 63°35′07,8″* *6. N 56°02′56,3″ E 63°35′07,8″* *7. N 56°02′55,9″ E 63°35′07,5″* *8. N 56°02′53,8″ E 63°35′06,0″* *9. N 56°02′54,4″ E 63°35′06,5″* *10. N 56°02′53,8″ E 63°35′09,0″* *11. N 56°02′54,1″ E 63°35′12,2″*
Data accessibility	*Data are with this article in the*supplementary table 1*.*
Related research article	*I. Semenkov, T. Koroleva. The spatial distribution of fractions and the total content of 24 chemical elements in soil catenas within a small gully's catchment area in the Trans Urals, Russia, Applied Geochemistry**2019**(106) 1 – 6.*

**Table:** 

**Value of the data** • Data could be used by other researchers for understanding migration and accumulation of different compounds in soil catenae. • Data could be used for the assessment of contamination level of potentially toxic elements. It will be more informative for scientists for their further research. • Data will be important for estimating health risk posed by potentially toxic element contamination in soil. • Data may be useful for policy makers to create health risk management plan. • Data obtained can be used for more confident identification of pollution sources and pollutants' migration routes and more effective conservation and remediation of human-affected soils within forest-steppe areas.

## 1 Data

The Trans Urals forest-steppe region has one of the highest population densities in Siberia and limited application of micro fertilizers. Geologically, the region is composed of Palaeogenic marine clays, sandstones, opokas and diatomites that are overlain by loess-like loams. Sloping plains of abrasive-erosive and alluvial-lacustrine genesis and Neogene-Quaternary ages with flat or slightly undulating interfluves of 110–140 m a.s.l. are deeply dissected by well-developed valleys of rivers [4]. According to the Köppen-Geiger classification, the area belongs to the snow fully humid type with warm summer conditions (Dfb) [5].

**Fig. 1:**
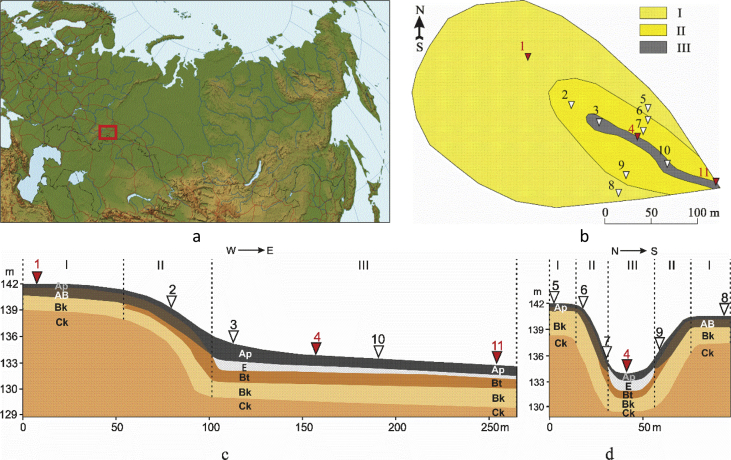
Sketch maps showing location of the catchment area (left) and soil pits (right) and sketches of soil profiles within the catenae (c – the catena along the gully and d – the catenas across the gully). Soil pits (numbers 1–11) are located in the following topographic locations: I – the levelled interfluve, II – slopes and III – the bottom of the U-shaped gully. In the pits marked in red, water extracts and mineralogical composition are analysed. Designations of soil horizons are according to [14].

**Fig. 2:**
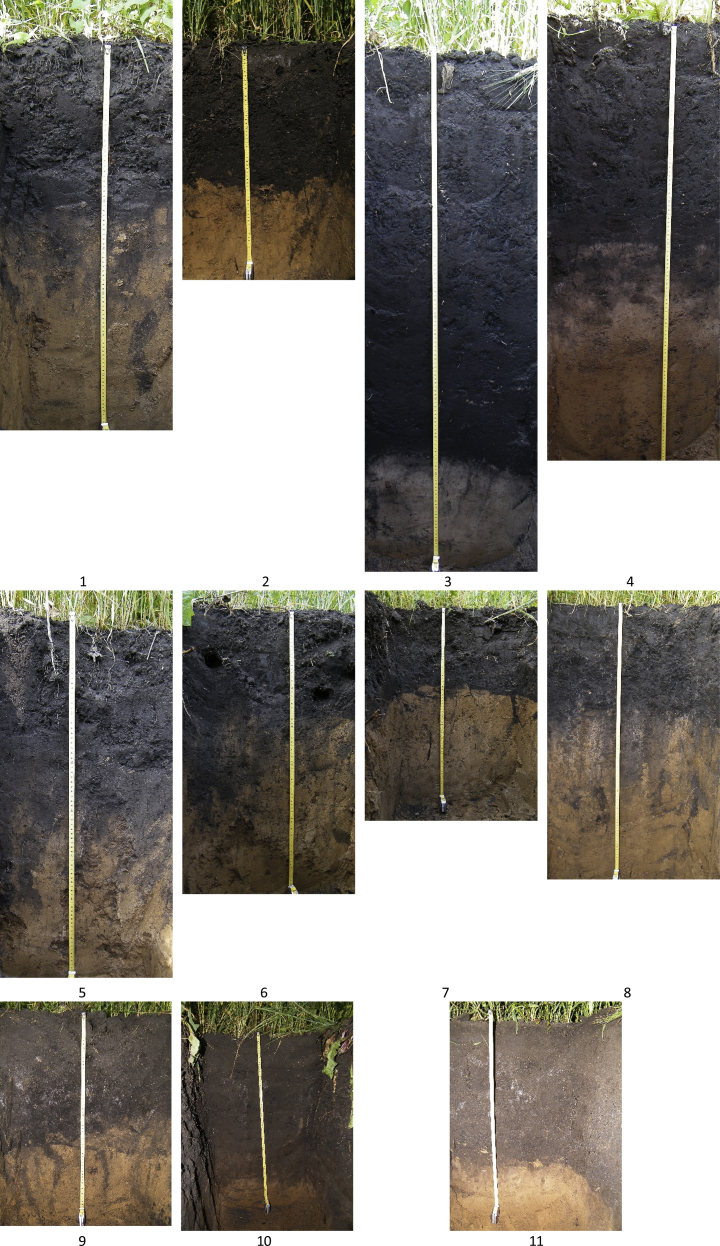
Photos of soil pits (numbers 1–11). Pit locations are shown in Fig. 1.

Data were collected within the catchment area (480 000 m^2^) of a U-shaped gully on the Trans-Urals Elevation of the Ishim Plain (Fig. 1). It was selected by means of analysing the maps of vegetation, soils, parent materials and geochemical migration factors within the Ob river basin area [6]. Within the catchment, Anthric Chernozems were found on interfluves and slopes and Anthric Planosols – at the bottom of the gully. This site represents a group of soil catenas typical for the central part of Western Siberia [7]. During the fieldwork, 60 soil samples (300–500 g) were collected from 11 soil pits (Fig. 1, Fig. 2, Table 1).

**Table 1:** Morphological properties of Anthric Chernozems and Anthric Planosols.

Soil	Horizon	Depth, cm	Colour description	Mansell	Structure
Chernozems	Ap	0–20	Uniform, dark grey	10YR 2/1	Angular blocky with some crumb-like peds
	A	20–30(40)	Uniform, dark grey	10YR 2/1	Small angular blocky or subangular blocky
	AB	30(40)–55(75)	Brown background with numerous dark grey, sharply delineated, vertically oriented stripes of humic material	10YR 7/4	Angular blocky and prismatic
	Bk	55(75) – 195	Brown with lighter mottles (CaCO_3_ concentrations)	10YR 6/6	Prism-like peds consisting of small angular block-like peds
	Ck	195–220	Uniform, brown	2.5Y 6/6	Structureless
Planosols	Ap	0–20	Uniform, dark grey	10YR 2/1	Angular/subangular blocky with some crumb-like peds
	A	20–80(100)	Uniform, dark grey	10YR 2/1	Angular/subangular blocky and crumb
	E	80(100) – 110(130)	Uniform, whitish grey	2,5Y 6/3	Laminated
	Bt	110(130) – 200	Uniform, brown	10YR (4–5)/6	Prism-like peds consisting of small angular block-like peds
	C	200–220	Uniform, brown	2.5Y 6/6	Structureless

Data included TOC, рН, particle-size distribution, total concentrations of 23 ChEs and content of three mobile fractions of 61 ChEs (Table S1). Electrical conductivity, basicity and concentration of anions and cations were determined in the upper part of plough layers and the main horizons of three soil pits (Fig. 1). Mineralogical composition was analysed in samples from the most typical pits of Anthric Chernozem on the interfluve and Anthric Planosol at the gully bottom (Table 2).

**Table 2:** Mineralogy of Anthric Chernozems and Anthric Planosols.

Soil	Horizon	Smectite	Illite	I/Sm	Kaolinite	Chlorite	Plagioclases	PFS	Quartz	Calcite
Chernozems	Ap	24,2	7,1	10,7	4,2	0,3	10,4	3,9	38,6	0,6
	Сk	21,7	5,8	5,7	4,7	1,1	8,8	10,6	40,4	1,1
Planosols	Ap	24,6	6,8	16,2	4,1	0,9	7,8	5,3	34,0	0,4
	E	7,5	9,1	8,0	2,7	1,4	14,7	7,9	48,0	0,6
	C	25,7	5,7	10,9	3,0	1,5	11,9	5,9	35,0	0,4

I/Sm – illite-smectite mixed-layer minerals with predomination of illite interlayers, PFS – potassium feldspars.

## 2 Experimental design, materials and methods

Soil samples were taken from 7 pits of Anthric Chernozems and 4 pits of Anthric Planosols (Table 1) and analysed by standard techniques.

At the Faculty of Geography of MSU (Lomonosov Moscow State University), total organic carbon (TOC) in soils, electrical conductivity, basicity (HCO_3_^−^) and pH of water extracts were determined by standard techniques [1], [2], [3]. The particle-size distribution in soils was analysed using a laser diffraction technique and an ‘Analizeter 22’ equipment (Germany). The particle-size classes were defined as: G1 – clay (particles <2 μm), G2 – fine silt (2–6.3 μm), G3 – medium silt (6.3–20 μm), G4 – coarse silt (20–63 μm), G5 – fine sand (63–200 μm), G6 – medium sand (200–630 μm) and coarse sand (630–2000 μm). Anions (Cl^−^, SO_4_^2−^, NO_3_^−^, PO_4_^3−^) and cations (Ca^2+^, Mg^2+^, K^+^, Na^+^, NH_4_^+^) in water extracts (soil:solution ratio of 1:5) were measured by high performance liquid chromatography using a Styer chromatograph (Aquilon, Russia).

Total content of chemical elements (ChEs) was measured at the IGEM RAS (Institute of Geology of Ore Deposits, Petrography, Mineralogy and Geochemistry of the Russian Academy of Sciences) using an Axios X-Ray fluorescence spectrometer (made by PANalytical, Netherlands) and Russian Soil Standard samples (SSs of ‘Chernozem’ and ‘Albeluvisol’) with accuracy reported in [7].

Mobile fractions F1 – F3 were obtained according to the extraction procedure by [8] with the use of the following reagents: F1 – with NH_4_Ac (ammonium acetate buffer) and the soil:solution ratio of 1:5, F2 – with 1% EDTA (ethylenediaminetetraacetic acid) and the soil:solution ratio of 1:5 and F3 – with 1M HNO_3_ and the soil:solution ratio of 1:10. Concentrations of the extracted ChEs in the filtrates were determined using an Elan-6100 ICP-MS System (Inductively Coupled Plasma Mass Spectrometer by PerkinElmer Inc., USA) and an Optima-4300 DV ICP-AES System (Inductively Coupled Plasma Atomic Emission Spectrometer by PerkinElmer Inc., USA) at the VIMS (N. M. Fedorovskii All-Russia Institute of Mineral Raw Materials). The procedure [8] is regularly used for analysing mobile fractions of ChEs in soils in Russia. The ChE mobility (ChEm) was calculated as a ratio of its mobile fractions (F1+F2+F3) to its total content, multiplied by 100%.

At the MSU Faculty of Geology, soil mineralogical composition (phyllosilicates and other minerals) was determined using an ULTIMA-IV X-Ray diffractometer (made by Rigaku, Japan) operated at 40 kV, 40 mA, 3–65^о^ 2θ, with Cu radiation and a DTex/Ultra semiconductor detector. Minerals were identified by comparing experimental data with standard X-Ray patterns from the PDF-2 database with the use of the MDI Jade-6.5 software and methodological recommendations by [9], [10], [11]. A quantitative mineralogical analysis was carried out using the Rietveld full-pattern fitting method [12] and the BGMN software [13].

Statistical analyses included calculations of percentiles, median, median absolute deviation, mode for topsoils and subsoils of Chernozems and Planosols. Moreover, the normal distribution hypothesis was tested on all parameters analysed (Table S2).

## References

[1] International Standard (2005). Soil Quality — Determination of pH, ISO 103902005.

[2] Reeuwijk L. (2002). Procedures for Soil Analysis.

[3] Kramer J.R. (1982). Alkalinity and acidity. Inorg. Species.

[4] Zemtsov A.A., Mizerov B.V., Nikolaev V.A. (1988). Relief Zapadno-Sibirskoi Nizmennosti.

[5] Peel M.C., Finlayson B.L., McMahon T.A. (2007). Updated world map of the Köppen-Geiger climate classification. Hydrol. Earth Syst. Sci..

[6] Semenkov I.N., Miroshnikov A.Y., Asadulin E.E., Usacheva A.A., Velichkin V.I., Laverov N.P. (2015). The Ob river basin as a source of Kara Sea contamination with global fallout of Cesium-137. Dokl. Earth Sci..

[7] Semenkov I.N., Koroleva T.V. (2019). The spatial distribution of fractions and the total content of 24 chemical elements in soil catenas within a small gully's catchment area in the Trans Urals, Russia. Appl. Geochem..

[8] Solov’ev G.A., Izrael Y.A., Rovinsky F.Y. (1989). The use of complex extracts to determine the available forms of trace elements in soils. Monit. Backgr. Environ. Pollut..

[9] Harris W., White N.G., Ulery A.L., Drees R. (2008). X-ray diffraction techniques for soil mineral identification. Methods Soil Anal..

[10] Moore D.M., Reynolds R.C. j. (1997). X-Ray Diffraction and the Identification and Analysis of Clay Minerals.

[11] Drits V.A., Kossovskaya A.G. (1990). Clay Minerals: Smectites and Mixed–Layer Minerals. https://docplayer.ru/81639690-V-a-dric-a-g-kossovskaya-glinistye-mineraly-smektity-smeshanosloynye-obrazovaniya.html.

[12] Bish D.L., Post J.E. (1993). Quantitative mineralogical analysis using the Rietveld full-pattern fitting method. Am. Mineral..

[13] Doebelin N., Kleeberg R. (2015). Profex: a graphical user interface for the Rietveld refinement program BGMN. J. Appl. Crystallogr..

[14] FAO/WRB (2014). World Reference Base for Soil Resources 2014.

